# Broad synergistic antiviral efficacy between a novel elite controller-derived dipeptide and antiretrovirals against drug-resistant HIV-1

**DOI:** 10.3389/fcimb.2024.1334126

**Published:** 2024-06-10

**Authors:** Federica Giammarino, Anders Sönnerborg, Rafael Ceña-Diez

**Affiliations:** ^1^Division of Infectious Diseases/ANA Futura, Department of Medicine Huddinge, Karolinska Institutet, Huddinge, Sweden; ^2^Department of Infectious Diseases, Karolinska University Hospital, Stockholm, Sweden; ^3^Division of Clinical Microbiology, Department of Laboratory Medicine, Karolinska Institutet, Huddinge, Sweden

**Keywords:** WG-am, ARV, combination, synergy, HIV, drug resistance, INSTI

## Abstract

**Introduction:**

The naturally occurring dipeptide Tryptophylglycine (WG) is enhanced in human immunodeficiency virus (HIV-1) infected Elite Controllers (EC). We have shown that this dipeptide has an anti-HIV-1 effect and evaluated now its synergistic antiretroviral activity, in combination with current antiretrovirals against multi-drug resistant HIV-1 isolates.

**Methods:**

Drug selectivity assay with WG-am and ARVs agains HIV-1 resistant isolates were carried out. Subsequently, two methods, Chou-Talalay’s Combination Index (CI) and ZIP synergy score (SS), were used to quantify the synergism.

**Results:**

WG-am had a moderate/strong synergism with the four tested antiretrovirals: raltegravir, tenofovir, efavirenz, darunavir. WG-am:TDF had strong synergism at ED50, ED75, ED90 (CI: <0.2) in isolates resistant to protease inhibitors or integrase strand inhibitors (INSTI), and a slightly less synergism in isolates resistant to non-nucleoside or nucleotide reverse transcriptase inhibitors. WG-am combined with each of the four drugs inhibited all drug-resistant isolates with over 95% reduction at maximum concentration tested. The highest selectivity indexes (CC50/ED50) were in INSTI-resistant isolates.

**Conclusion:**

Our data suggest that WG, identified as occurring and enhanced in Elite Controllers has a potential to become a future treatment option in patients with HIV-1 strains resistant to any of the four major categories of anti-HIV-1 compounds.

## 1 Introduction

Despite major advances in antiretroviral therapy (ART), people living with human immunodeficiency virus type 1 (HIV-1) (PLWH) may fail to achieve viral suppression due to acquired drug resistance and an increasing spread of drug-resistant HIV-1 (Andersson et al., 2021). While newer generations of anti-HIV-1 drugs have a higher genetic barrier to resistance, the resistance mutations against the highly effective integrase strand transfer inhibitors (INSTIs) can develop (Andersson et al., 2021; Beesham et al., 2022; Li et al., 2022). Also, a subset of PLWH with extensive ART exposure has limited or exhausted treatment options also due to drug toxicity, drug–drug interactions, and/or comorbidities. Therefore, there is a need to develop new antiretroviral agents that can overcome these hurdles.

The dipeptide WG-am emerged as notably elevated in elite controllers (ECs) in comparison to viral progressors (VPs) and healthy controls (HCs). We have demonstrated the low toxicity of WG-am both in isolation and in combination with other compounds (Sperk et al., 2021; Cena-Diez et al., 2022). Building on these findings, we have shown that it has anti-HIV-1 activity (Sperk et al., 2021; Cena-Diez et al., 2023a). WG-am binds to the CD4 binding pocket of HIV-1 gp120 blocking the interaction between the virus and the CD4 receptor and also inhibits HIV-1 reverse transcription (Cena-Diez et al., 2023a). This dual antiviral mechanism and the low toxicity of WG-am suggest that it has the potential to be used against drug-resistant HIV-1.

Previously, we described the low toxicity and synergistic profile of WG-am and another novel antiviral compound, ssON, against drug-resistant HIV-1 (Cena-Diez et al., 2022; Cena-Diez et al., 2023b). To further study the efficacy of WG-am in combination with approved antiretrovirals, we now investigated its anti-HIV-1 activity combined with four antiretroviral agents from four classes: the protease inhibitor (PI) darunavir (DRV), the INSTI raltegravir (RAL), the nucleotide reverse transcriptase inhibitor (NRTI) tenofovir disoproxil fumarate (TDF), and the non-nucleoside RTI (NNRTI) efavirenz (EFV). The synergistic antiviral activity was evaluated against 28 drug-resistant HIV-1 isolates.

Our results demonstrated that the combination of WG-am and each of the four antiretrovirals had a strong synergistic profile in the majority of HIV-1 isolates resistant to each of the four major drug classes. We propose WG-am to be a viable complement to existing HIV-1 therapies, particularly in the context of drug-resistant HIV strains.

## 2 Materials and methods

### 2.1 Compounds

The amide form of tryptophylglycine (WG) was purchased from Pepscan (Lelystad, Netherlands) with a purity of >95%. TDF, RAL, EFV, and DRV were purchased from Selleckchem (Houston, USA). TDF, RAL, EFV, and DRV were initially dissolved in DMSO to 150 mM, 200 mM, 200 mM, and 180 mM, respectively, followed by dilution in phosphate-buffered saline (PBS)/DMSO to a final concentration for the stocks of 1 mM and a maximum of 10% DMSO. WG-am was dissolved in PBS.

### 2.2 Cells and viruses

The TZM.bl cell line (NIH AIDS Research and Reference Reagent Program, USA) and HEK-293T cells (ATCC, USA) were cultured in DMEM (Sigma, USA), supplemented with 10% FBS, penicillin/streptomycin (100 IU/mL and 50 mg/mL, respectively), and 2 mM of L-glutamine. Peripheral blood mononuclear cells (PBMCs) were isolated on a Ficoll–Paque Plus density gradient (Merk S.L., Madrid, Spain). Prior to treatment with the antiviral compounds, PBMCs were stimulated with the mitogen phytohemagglutinin (PHA) for 48 h (2 µg/mL; Thermo Fisher Scientific, Waltham, MA, USA).

Viral stocks of HIV-1 JRFL laboratory strain and drug-resistant isolates (DRIs) (**Supplementary Table 1**) were obtained by transient transfection of their corresponding plasmids (NIH AIDS Research and Reference Reagent Program) in HEK-293T cells (ATCC, Manassas, VA, USA). Supernatants were collected at 48 h and 72 h. Viral stocks were clarified by centrifugation prior to evaluating the viral titer by HIV-1 p24gag ELISA kit (INNOTEST^®^ Innogenetics, Belgium). Viral infectivity was assessed by tissue culture infective doses (TCID50) using the Spearman–Kärber method based on the results from the HIV-1 p24gag ELISA kit (Lei et al., 2021).

### 2.3 Drug sensitivity assay

#### 2.3.1 Drug-sensitive isolates

Drug sensitivity assays (DSAs) were performed to determine the CI between WG-am and RAL, TDF, DRV, or EFV against the reference virus JRFL. The drugs were serially diluted, ranging from 1 mM to 1 µM for WG-am and 1 µM to 1 nm for RAL, TDF, DRV, and EFV, and then added in triplicate in 96-well plates that had been seeded 24 h before the start with 10^4^ TZM-bl cells/well. The viruses were added to each well at 100 TCID50/well. After 48 h, viral infection was measured by luciferase assay. The ratios used for combining WG-am with the different antiviral drugs were calculated based on their non-toxic concentrations (**Supplementary Figure 1**). A ratio of 1,000:1 means that the first compound has a concentration 1,000 times higher than the second one.

#### 2.3.2 Drug-resistant isolates

Using the same protocol, the antiviral activity of WG-am alone and in combination with RAL, TDF, DRV, or EFV was tested against 28 DRIs. We evaluated RAL, TDF, EFV, and DRV, respectively, to confirm that the produced isolates were in fact resistant to the defined antiretroviral (ARV). The drug concentrations required to inhibit virus replication by 50% (ED50) were calculated from a dose–response curve using non-linear regression analysis (GraphPad Prism, version 8.0.1). The combination profiles of the compounds were determined using CalcuSyn software (Biosoft, Cambridge, UK), based on the median effect principle (Chou and Talalay, 1984). In Chou–Talalay’s, CI > 1.3 indicates antagonism, CI = 1.1–1.3 moderate antagonism, CI = 0.9–1.1 additive effect, CI = 0.8–0.9 slight synergism, CI = 0.6–0.8 moderate synergism, CI = 0.4–0.6 synergism, and CI = 0.1–0.4 strong synergism (Chou, 2010). However, since the support to the CalcuSyn software has been removed by Biosoft since December 2024, its use will be limited in the future.

Also, SynergyFinder 3.0 software was used to calculate additional SSs between WG-am and the ARV. Among the different models available, we selected the ZIP additivity method for our analysis since it integrates the advantages of the Loewe additivity model, which is frequently preferred for the comparison of two drugs with a similar mechanism of action, and the Bliss model, which computes the drug–drug interaction effect with respect to a multiplicative effect between two drugs acting independently from each other (Ianevski et al., 2022). We used a four-parameter logistic regression (LL4) as the default option for a curve-fitting algorithm, which is used to fit single-drug dose–response curves. However, some combinations required another curve-fitting algorithm; therefore, we used linear regression (LM) as an alternative. Heatmaps representing the SSs, inhibitory values, and clustering of the combinations were generated for each set of drug-resistant isolates using SynergyFinder 3.0 post-analysis options. In the ZIP method, SS > 10 indicates synergism, SS = 10 to −10 additive, and SS < −10 antagonism. In addition, the most synergistic area (MSA) score, which represents the most synergistic 3 × 3 dose window in a dose–response matrix, was calculated.

To fully evaluate the synergy of the compounds, the drug combinations were tested under various concentrations, in a so-called dose–response matrix design. Briefly, WG-am was tested from 1 mM and subsequent 1-fold dilutions until 1 µM, and one extra 1/2 dilution giving a concentration of 500 µM was added. For the ARV, a similar approach was followed with different starting concentrations of RAL (1 µM), TDF (10 µM), DRV (100 nM), and EFV (1 µM). Then, the dose–response matrix for each combination of WG-am/ARV was designed to include all possible dose combinations for the drug pair tested. The DSA experiments were performed with three technical replicates for each virus with the specified dynamic concentration range for each drug, which ranged from 0% to 100% inhibition. At least three independent analyses (biological replicates) were performed. The reproducibility of the DSA was assessed based on the 95% CI obtained for the drug ED50 and the degree of correlation between technical replicates.

### 2.4 Statistical analysis

Statistics were presented as means ± standard deviation (SD) unless otherwise noted. Parametric and/or non-parametric tests used are listed in the figure legends and tables. Statistical significance was accepted when *P* < 0.05. Analysis was performed using GraphPad Prism 8.0.1 (GraphPad, Inc.) and CalcuSyn (Biosoft).

## 3 Results

### 3.1 Combinations of WG-am:ARV have synergistic activity against drug-sensitive HIV-1 JFRL

**Figure 1:**
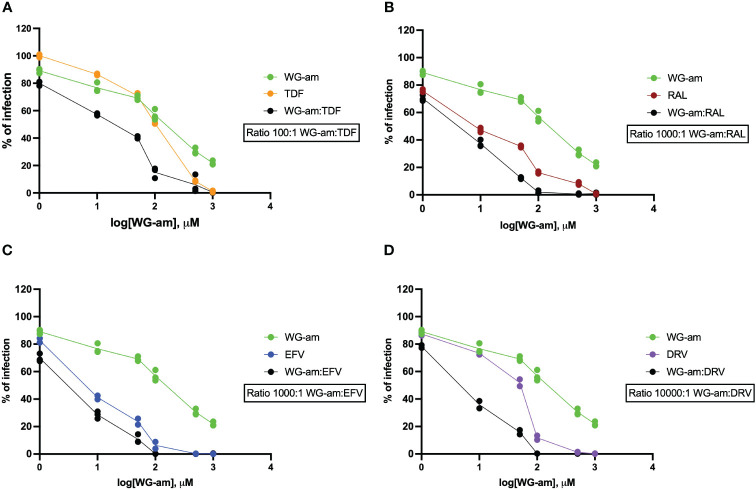
Drug susceptibility assays (DSAs) of the combinations of raltegravir (RAL, 1 nM to 1 μM), tenofovir (TDF, 10 nM to 10 μM), darunavir (DRV, 0.1 nM to 0.1 μM), or efavirenz (EFV, 1 nM to 1 μM) with WG-am: **(A)** WG-am:TDF, **(B)** WG-am:RAL, **(C)** WG-am:EFV, and **(D)** WG-am:DRV. The DSA was performed in TZM.bl against the wild-type prototype JRFL isolate. The percentage of viral infection was determined at 48 h by quantification of luciferase expression levels. Data were plotted as mean ± standard deviation of three different experiments, and NT values were used as 100% of infection for each isolate.

WG-am had synergism with all ARVs (RAL, TDF, EFV, DRV), at least in one drug concentration, against a drug-sensitive JFRL isolate (**Table 1**, **Figure 1**). All combinations reached 100% inhibition at low concentrations. A strong synergism was found with DRV (0.264) and RAL (0.253), respectively, at the highest effective dose (ED90). A moderate synergism was found with EFV (0.628) and TDF (0.623). Notably, WG-am:TDF at 100:1 ratio had a higher ED50 concentration (ED50: 11.404 μM) compared to WG-am:RAL (3.522 μM), WG-am:EFV (2.385 μM) (both at 1,000:1 ratio), and WG-am:DRV (3.717 μM) (at 10,000:1 ratio) (**Table 1**).

**Table 1:** Combination index (CI) and ED50 calculations of WG-am with tenofovir (TDF), raltegravir (RAL), efavirenz (EFV), or darunavir (DRV).

Virus	Drug combination	Ratio	Combination index values at			ED50 (µM WG-am)
			ED50	ED75	ED90	
JRFL	WG-am:TDF	100:1	0.193	0.288	0.623	11.404
JRFL	WG-am:RAL	1,000:1	0.525	0.363	0.253	3.542
JRFL	WG-am:EFV	1,000:1	0.353	0.462	0.628	2.385
JRFL	WG-am:DRV	10,000:1	0.296	0.271	0.264	3.717

The combinations were prepared using a certain dose gradient (at least five doses) according to the ratio stipulated in the table, using a wild-type prototype JRFL isolate. The CI values at different effective doses (EDs) using the CalcuSyn software are shown. CI > 1.3 indicates antagonism, CI = 1.1 to 1.3 moderate antagonism, CI = 0.9 to 1.1 additive effect, CI = 0.8 to 0.9 slight synergism, CI = 0.6 to 0.8 moderate synergism, CI = 0.4 to 0.6 synergism, and CI = 0.1 to 0.4 strong synergism. ED50 values were calculated from a dose–response curve using non-linear regression analysis (GraphPad Prism, version 8.0.1; GraphPad Software, La Jolla, CA, USA).

### 3.2 Combinations of WG-am:ARV have synergistic antiviral activity against drug-resistant isolates

Next, we evaluated the antiviral profiles using drug-resistant HIV-1 isolates. The results were expressed as CI, ED50, and ZIP SS. The resistance reported by NIH was confirmed for all viral isolates by our DSA (**Supplementary Table 1** and **Heatmaps 1 to 4**).

#### 3.2.1 Effects on INSTI- and PI-resistant isolates

The WG-am:ARV combinations were used at the same ratios as the drug-sensitive isolate. WG-am:TDF showed the strongest synergism (at ED75 and ED90) against all isolates that had resistance to PI (ED90: 0.124) and INSTI (ED90: 0.05) compared to the combinations with RAL, EFV, and DRV (**Table 2** and **Supplementary Tables 2**, **3A, B**). In addition, ZIP SS showed synergy or at least a strong additivity effect (**Supplementary Table 2** and **Heatmap 1**).

**Table 2:** WG-am:ARV combinations against a selection of the drug-resistant isolates tested.

Isolate	Drug resistance	ARV	Ratio WG-am:ARV	Average combination index values at			ED50 (µM WG-am)	ZIP synergy score
				ED50	ED75	ED90		
**4736_4**	INSTI	RAL	1,000:1	0.403	0.363	0.343	15.081	−4.79
**4736_4**	INSTI	EFV	1,000:1	0.455	0.339	0.266	2.122	2.33
**4736_4**	INSTI	TDF	100:1	0.269	0.064	0.018	6.275	0.39
**4736_4**	INSTI	DRV	10,000:1	0.239	0.176	0.142	3.797	1.92
**8070_1**	INSTI	RAL	1,000:1	0.402	0.96	1.301	24.599	−5.92
**8070_1**	INSTI	EFV	1,000:1	0.787	0.699	0.682	3.902	4.77
**8070_1**	INSTI	TDF	100:1	0.303	0.116	0.063	6.054	1.00
**8070_1**	INSTI	DRV	10,000:1	0.267	0.245	0.2464	2.658	6.10
**CA96453**	PI	RAL	1,000:1	0.400	0.184	0.086	3.299	8.27
**CA96453**	PI	EFV	1,000:1	0.578	0.464	0.416	3.074	8.75
**CA96453**	PI	TDF	100:1	0.387	0.209	0.147	9.150	−3.54
**CA96453**	PI	DRV	10,000:1	0.360	0.203	0.137	4.933	1.04
**CA126796**	PI	RAL	1,000:1	0.204	0.076	0.028	2.169	7.02
**CA126796**	PI	EFV	1,000:1	0.365	0.316	0.301	2.283	4.75
**CA126796**	PI	TDF	100:1	0.353	0.166	0.117	8.726	−0.82
**CA126796**	PI	DRV	10,000:1	0.310	0.244	0.295	5.101	2.39
**7324–1**	NRTI	RAL	1,000:1	0.636	0.128	0.027	3.717	5.07
**7324–1**	NRTI	EFV	1,000:1	0.353	0.191	0.122	1.693	8.85
**7324–1**	NRTI	TDF	100:1	0.351	0.164	0.077	11.529	1.14
**7324–1**	NRTI	DRV	10,000:1	0.191	0.133	0.133	3.622	5.12
**4755–5**	NRTI	RAL	1,000:1	0.456	0.131	0.040	3.508	8.06
**4755–5**	NRTI	EFV	1,000:1	0.471	0.233	0.133	3.134	8.85
**4755–5**	NRTI	TDF	100:1	0.321	0.048	0.080	6.144	8.25
**4755–5**	NRTI	DRV	10,000:1	0.240	0.197	0.201	3.470	5.67
**5485**	NNRTI	RAL	1,000:1	0.528	0.130	0.033	4.914	3.15
**5485**	NNRTI	EFV	1,000:1	0.233	0.136	0.101	7.411	2.53
**5485**	NNRTI	TDF	100:1	0.315	0.111	0.056	5.184	7.01
**5485**	NNRTI	DRV	10,000:1	0.220	0.169	0.204	3.140	3.04
**25641**	NNRTI	RAL	1,000:1	0.295	0.165	0.093	2.959	6.59
**25641**	NNRTI	EFV	1,000:1	0.310	0.136	0.026	5.540	3.66
**25641**	NNRTI	TDF	100:1	0.321	0.098	0.031	5.886	9.92
**25641**	NNRTI	DRV	10,000:1	0.271	0.170	0.137	2.654	3.36

The combination index (CI), ED50, and ZIP SS calculations of WG-am in combination with TDF and DRV against isolates with resistance to integrase strand transfer inhibitors (INSTIs), protease inhibitors (PIs), nucleotide reverse transcriptase inhibitor (NRTI), and non-nucleoside reverse transcriptase inhibitor (NNRTI). The CI at different effective doses (EDs) was determined using the CalcuSyn software. The ZIP SS was calculated using SynergyFinder 3.0 software. The full information is provided in **Supplementary Tables 2–5**.

WG-am:RAL showed moderate synergism with two of the INSTI-resistant isolates but an additive effect at ED75 (0.96) and low antagonist effect at ED90 (1.301) using the third INSTI-resistant isolate 8070_1. The antagonism against isolate 8070_1 could be related to the highest RAL concentration used (5 μM), which usually completely inhibits wild-type HIV-1 *in vitro*, but not these highly resistant isolates. Similarly, ZIP SS reported an additive effect of WG-am with RAL against the INSTI-resistant isolates 4736_2 (−4.79), 8070_1 (−5.91), and 4736_4 (−4.2) (**Supplementary Table 2** and **Heatmap 1**). Despite these discrepancies between CalcuSyn and SynergyFinder 3.0. data, WG-am:RAL inhibited the infection over 90% with the highest ED50 of all combinations and isolates (ED50: 15–26 μM WG-am) (**Supplementary Table 2**). WG-am:EFV inhibited all INSTI- and PI-resistant isolates showing a moderate/strong synergism with ED90 ranging from 0.682 to 0.147 (IQR: 0.0485) for different resistant isolates (ED90 0.682–0.147) (**Supplementary Tables 2**, **3**).

WG-am:DRV had a moderate/strong synergism against all 11 PI-resistant isolates (ED90 0.066–0.349, IQR: 0.1655) with 100% inhibition (**Supplementary Tables 3A, B**). WG-am:DRV concentration averaged a low ED50 of 7.508 μM WG-am and 0.7508 nM DRV (IQR: 3.86). Also, using ZIP SS, all WG-am:ARV combinations had synergy or at least additive effect, against all PI-resistant isolates (**Heatmap 2**). However, Chou–Talalay’s algorithm reported that these combinations were synergistic at high inhibitory concentrations against these isolates (ED90 0.147 and 0.122, respectively).

When we analyzed the heatmap generated for PI-resistant isolates (**Heatmap 2**), WG-am:DRV and WG-am:TDF did not have synergy and only an additive effect (both the antivirals did not inhibit the infection at all >10% for the first two concentrations tested), but a strong synergism was obtained at high concentrations achieving 100% inhibition.

#### 3.2.2 Effects on RTI-resistant isolates

All combinations showed a synergistic profile against the five NRTI-resistant isolates at ED50, ED75, and ED90 (**Table 2** and **Supplementary Table 4**). ZIP SS also reported these combinations as synergistic for WG-am:EFV and WG-am:RAL (**Heatmap 3**). Similar to the PI-resistant isolates, WG-am:DRV and WG-TDF ZIP SS only reported an additive effect. The MSA of these combinations reported a synergism at high concentrations in 4/5 isolates with DRV and 3/5 with TDF. The combination of WG-am:TDF did show a lower SS with 525,342 (SS: −2.91 and MSA: 1.77). WG-am:TDF combination showed the highest ED50 (9.89 μM WG-am/98.9 nM TDF) of all combinations.

To study NNRTI drug-resistant isolates, we generated two separate heatmaps, one for WG-am:RAL, WG-am:EFV, and WG-am:TDF using the LL4 curve-fitting algorithm (**Heatmap 4**) and one for WG-am:DRV using the LM curve-fitting algorithm (**Heatmap 5**). WG-am:DRV did not exhibit a dose–response sigmoidal (S-shaped) curve needed for the LL4 algorithm; therefore, the ZIP SS calculated was not correct (**Heatmap 4**). We decided to use the LM algorithm for WG-am:DRV to study and visualize the data for NNRTI-resistant isolates and to use WG-am:TDF as control (**Heatmap 5**). As we can see in **Table 2** and **Supplementary Tables 5A, B**, WG-am:EFV, WG-am:TDF, and WG-am:RAL combinations had a very strong synergism at the high inhibitory concentrations ED75 (0.162, 0.095, 0.121) and ED90 (0.062, 0.071, 0.076). WG-am:DRV showed a strong synergism at ED50, ED75, and ED90. ZIP SS showed an additive with the majority showing synergistic MSA (**Table 2** and **Supplementary Tables 5B** and **Heatmap 5**). It showed no synergism at low concentrations at which DRV had no effect against these resistant isolates but strong synergism at high effective doses (ED90).

The ED50 for the WG-am combination with RAL, EFV, TDF, and DRV ranged from 2.9 to 4.91 μM/nM, 2.70 to 9.98 μM/nM, 4.90/49.0 to 7.21/72.1 μM/nM, and 1.93/0.193 to 3.97/0.397 μM/nM, respectively (**Supplementary Tables 5A, B**).

## 4 Discussion

The existence of highly treatment-experienced patients with few remaining therapeutic options and the spread of drug-resistant HIV-1 illustrate the need to develop new anti-HIV-1 drugs despite the great success of ART in most patients (Clutter et al., 2016; Biswas et al., 2019; Chen et al., 2020). Here, we describe that our novel compound WG-am has synergistic effects with four antiretrovirals—RAL, TDF, DRV, and EFV—using drug-sensitive and drug-resistant isolates. The synergistic effects were stronger with the INSTI RAL and the PI DRV, although synergy was seen also for TDF (NRTI) and EFV (NNRTI) (**Table 1**). Our data suggest the value of combining WG-am with drugs of any class.

Among the mix of factors that contribute to the development of an elite control profile, the dipeptide WG-am stands out for being capable of inhibiting HIV-1 infection on its own. The dual mechanism of WG-am makes it an interesting candidate for combination with antiretrovirals from different families, especially targeting multiresistant HIV-1. Thus, the mechanisms give it a higher threshold for development of mutations at which the virus can escape its inhibition. Furthermore, the fact that it functions as both an entry and RT inhibitor allows it to have a synergistic profile with all the antivirals analyzed regardless of their mode of action. The dual-action mechanism of WG-am manifests a synergistic effect when combined with the four tested compounds, notwithstanding their diverse mechanisms of action. WG-am’s first mechanism of action is entry inhibition, which is distinct from the mechanisms of the tested ARVs. We have earlier shown that WG-am does not act after the RT step. Therefore, the lack of antagonistic effect between WG-am and the INSTI raltegravir and the PI darunavir is not unexpected. However, such an effect could possibly be anticipated in our examination due to the interaction between WG-am and the RTIs TDF and EFV in the RT step. Despite this anticipation, our findings demonstrated a notable synergistic relationship between WG-am and these compounds. Previously, we showed a synergy of WG-am and TDF against NRTI-resistant isolates 7324–1 and 52534–2 using the CalcuSyn software (Cena-Diez et al., 2022; Cena-Diez et al., 2023b). However, in the current manuscript, we expanded this finding and now report a synergy extended to all isolates exhibiting resistance against NNRTIs and NRTIs. These results are in line with our earlier experimental data hypothesizing that WG-am might act on the early steps of RT with a mechanism different from the NRTI and NNRTI. This observation highlights the promising potential of WG-am in addressing resistance issues while amplifying the therapeutic efficacy of existing ARVs.

Newer INSTIs—dolutegravir (DTG), cabotegravir (CAB), and bictegravir (BIC)—are cornerstones in modern ART. However, resistance may still develop, and there is a cross-resistance to the first-generation INSTI [RAL, elvitegravir (EVG)] (Duarte et al., 2022). The high efficacy of WG-am:EFV, WG-am:TDF, and WG-am:DRV on isolates resistant to INSTIs could make such WG-am combinations attractive options in patients failing regimens with INSTI resistance if WG-am is further developed and shown to hold through in clinical trials. Also, resistance to CAB develops somewhat more often in HIV-1 subtype A6 strains (Hu et al., 2023), wherein resistance to the NNRTI rilpivirine is present. Therefore, the efficacy of WG-am on all subtypes, earlier reported by us (Cena-Diez et al., 2022), may be relevant in such patients.

All combinations showed a strong synergism against the two INSTI-resistant isolates 4736_2 and 4736_4, which have 13 common drug resistance mutations, e.g., including N155H which reduces RAL and EVG susceptibility approximately 10- and 30-fold, respectively, and contributes to reduced susceptibility to DTG and CAB (Rhee et al., 2019). The 8070–1 isolate had three major INSTI-resistant mutations (G140S, Y143H, Q148H) giving resistance to RAL as well as DTG and CAB. G140S in combination with Q148 confers >100-fold reduced susceptibility to RAL, 10-fold reduced susceptibility to CAB, and 2- to 5-fold reduced susceptibility to DTG (Yoshinaga et al., 2015; Zhang et al., 2018; Saladini et al., 2019). Nonetheless, all our drug combinations completely inhibited the infection with these INSTI-resistant isolates. It can be noted that the WG-am:RAL inhibited the infection by more than 95% in all isolates despite high resistance to RAL (>100-fold reduced activity).

In addition, all WG-am combinations had a strong synergy or at least an additive effect (ZIP SS), against all PI-, NRTI-, and NNRTI-resistant isolates, except WG-am:DRV against PI-resistant isolates CA96454 and CA96453 (major mutations 46I and 90M), respectively, which showed a weak additive using ZIP SS. However, in both cases, a strong synergism was found using Chou–Talalay’s method at all effective doses >50% (**Supplementary Tables 5A, B**). When we used post-analysis tools of these results using SynergyFinder 3.0, we showed that the absence of synergism was due to the lack of synergy at low concentrations despite the strong synergism found at high inhibitory concentrations.

One of the antiviral mechanisms reported for WG-am is inhibition of HIV-1 RT (Cena-Diez et al., 2023a). Our data support that WG-am can be combined with NRTI and NNRTI inhibitors, like EFV or TDF, against RTI-resistant isolates. Also, WG-am itself was able to completely inhibit NRTI- and NNRTI-resistant isolates.

The usage of two different software programs, CalcuSyn and SynergyFinder 3.0, to calculate the efficacy of the drug combinations increases the strength of our data. CalcuSyn utilizes Chou–Talalay’s method, providing ED50, ED75, and ED90 values, for different inhibitory areas thereby offering insights into drug combination efficacy at various concentrations. SynergyFinder 3.0 employs the ZIP synergy method and stands out for its intuitive visualization and post-analysis tools. None out of the 112 comparisons, commented above, were reported as synergistic by Chou–Talalay’s method and antagonistic by SynergyFinder 3.0. In addition, 85 out of 112 analyses were reported as additive and the rest as synergistic by ZIP SS. However, almost half of those (34/85) that were reported as additives had an MSA that indicated that they presented a synergistic profile at high inhibitory concentrations. On the other hand, Chou–Talalay’s algorithm divides the calculation into sections depending on the inhibition obtained (ED50, ED75, ED90). Since the intention is to inhibit the infection completely, we suggest that for the purpose of our study, the CI value ED90 provided by Chou–Talalay’s software could be more relevant.

The absence of synergy reported by the ZIP method at low concentrations could potentially be important. Ideally, it is desired to obtain synergy at low concentrations of the compounds in order to diminish toxicity problems. However, in the present analysis, also the higher concentrations evaluated are well below the maximum non-toxic concentrations (**Supplementary Figure 1**). Furthermore, the intention of combination therapy is to completely inhibit the replication; therefore, the presence of synergy in more than 70% of the combinations against the different isolates resistant to antiretrovirals at high inhibitory concentrations (>90%) is of great relevance.

The observed naturally enhanced presence of WG in EC, seemingly without associated toxicity or adverse effects, highlights a potential correlation between elevated WG-am levels and disease control. While our study does not conclusively attribute elite controller status to this dipeptide, it underscores the need for further investigation into their specific biological role, both in persons with or without HIV-1. Understanding the precise mechanism by which WG-am functions and its potential impact on overall human health is imperative. Our findings suggest a promising avenue for the inclusion of WG-am in future therapeutic strategies; however, rigorous preclinical and early phase I clinical studies are warranted to comprehensively evaluate its biocompatibility, safety profile, and potential integration into existing antiretroviral combination therapies.

In conclusion, our *in-vitro* results show that WG-am has the potential to become a novel antiretroviral agent that can overcome HIV-1 drug resistance against other classes of antiretrovirals, exhibiting synergistic activity with commonly used antiretroviral agents. This makes WG-am an attractive candidate for further development as a new anti-HIV-1 drug.

## Data availability statement

The original contributions presented in the study are included in the article/**Supplementary Material**. Further inquiries can be directed to the corresponding author.

## Ethics statement

Ethical approval was not required for the studies on humans in accordance with the local legislation and institutional requirements because only commercially available established cell lines were used.

## Author contributions

FG: Data curation, Investigation, Writing – review & editing. AS: Conceptualization, Funding acquisition, Project administration, Resources, Supervision, Visualization, Writing – review & editing. RC: Conceptualization, Data curation, Formal analysis, Funding acquisition, Investigation, Methodology, Software, Supervision, Validation, Writing – original draft, Writing – review & editing.

## References

[B1] Andersson E. Ambikan A. Brannstrom J. Aralaguppe S. G. Yilmaz A. Albert J.. (2021). High-throughput sequencing reveals a high prevalence of pretreatment HIV-1 drug resistance in Sweden. AIDS 35, 227–234. doi: 10.1097/QAD.0000000000002740 33394670

[B2] Beesham I. Parikh U. M. Mellors J. W. Joseph Davey D. L. Heffron R. PALANEE-Phillips T.. (2022). High levels of pretreatment HIV-1 drug resistance mutations among South African women who acquired HIV during a prospective study. J. Acquir. Immune Defic. Syndr. 91, 130–137. doi: 10.1097/QAI.0000000000003027 36094478 PMC9651927

[B3] Biswas A. Haldane A. Arnold E. Levy R. M. (2019). Epistasis and entrenchment of drug resistance in HIV-1 subtype B. Elife 8, e50524. doi: 10.7554/eLife.50524.030 31591964 PMC6783267

[B4] Cena-Diez R. Narayanan A. Ray S. van de Klundert M. Rodriguez J. E. Nilvebrant J.. (2023a). Naturally occurring dipeptide from elite controllers with dual anti-HIV-1 mechanism. Int. J. Antimicrob. Agents 61, 106792. doi: 10.1016/j.ijantimicag.2023.106792 36931610

[B5] Cena-Diez R. Singh K. Spetz A. L. Sonnerborg A. (2022). Novel naturally occurring dipeptides and single-stranded oligonucleotide act as entry inhibitors and exhibit a strong synergistic anti-HIV-1 profile. Infect. Dis. Ther. 11, 1103–1116. doi: 10.1007/s40121-022-00626-8 35391633 PMC9124260

[B6] Cena-Diez R. Spetz A. L. Sonnerborg A. (2023b). Synergistic antiviral activity against drug-resistant HIV-1 by naturally occurring dipeptide and A single-stranded oligonucleotide. Drug Resist. Update 68, 100955. doi: 10.1016/j.drup.2023.100955 36878096

[B7] Chen J. Liu Y. Liu S. Yuan D. Su L. Ye L.. (2020). HIV-1 drug resistance, distribution of subtypes, and drug resistance-associated mutations in virologic failure individuals in Chengdu, Southwest China 2014–2016. BioMed. Res. Int. 2020, 5894124. doi: 10.1155/2020/5894124 32280691 PMC7128060

[B8] Chou T. C. (2010). Drug combination studies and their synergy quantification using the Chou-Talalay method. Cancer Res. 70, 440–446. doi: 10.1158/0008-5472.CAN-09-1947 20068163

[B9] Chou T. C. Talalay P. (1984). Quantitative analysis of dose-effect relationships: the combined effects of multiple drugs or enzyme inhibitors. Adv. Enzyme Regul. 22, 27–55. doi: 10.1016/0065-2571(84)90007-4 6382953

[B10] Clutter D. S. Jordan M. R. Bertagnolio S. Shafer R. W. (2016). HIV-1 drug resistance and resistance testing. Infect. Genet. Evol. 46, 292–307. doi: 10.1016/j.meegid.2016.08.031 27587334 PMC5136505

[B11] Duarte F. C. Moura L. M. Laranjinha J. (2022). High-level dolutegravir resistance can emerge rapidly from few variants and spread by recombination: implications for INSTI salvage therapy. AIDS 36, 1881–1882. doi: 10.1097/QAD.0000000000003326 36172870

[B12] Hu Z. Cordwell T. Nguyen H. Li J. Jeffrey J. L. Kuritzkes D. R. (2023). Effect of the L74I polymorphism on fitness of cabotegravir-resistant variants of human immunodeficiency virus 1 subtype A6. J. Infect. Dis. 228, 1352–1356. doi: 10.1093/infdis/jiad291 37497681

[B13] Ianevski A. Giri A. K. Aittokallio T. (2022). SynergyFinder 3.0: an interactive analysis and consensus interpretation of multi-drug synergies across multiple samples. Nucleic Acids Res. 50, W739–W743. doi: 10.1093/nar/gkac382 35580060 PMC9252834

[B14] Lei C. Yang J. Hu J. Sun X. (2021). On the calculation of TCID(50) for quantitation of virus infectivity. Virol. Sin. 36, 141–144. doi: 10.1007/s12250-020-00230-5 32458296 PMC7973348

[B15] Li C. L. Liang H. Y. Xiao J. Li R. Yu F. T. Zeng Y. Q.. (2022). The effect of pretreatment potential resistance to NNRTIs on antiviral therapy in patients with HIV/AIDS. J. Acquir. Immune Defic. Syndr. 91, S27–S34. doi: 10.1097/QAI.0000000000003039 36094512

[B16] Rhee S. Y. Grant P. M. Tzou P. L. Barrow G. Harrigan P. R. Ioannidis J. P. A.. (2019). A systematic review of the genetic mechanisms of dolutegravir resistance. J. Antimicrob. Chemother. 74, 3135–3149. doi: 10.1093/jac/dkz256 31280314 PMC6798839

[B17] Saladini F. Giannini A. Boccuto A. Dragoni F. Appendino A. Albanesi E.. (2019). Comparable *in vitro* activities of second-generation HIV-1 integrase strand transfer inhibitors (INSTIs) on HIV-1 clinical isolates with INSTI resistance mutations. Antimicrob. Agents Chemother. 64 (1), e01717-19. doi: 10.1128/AAC.01717-19 31611362 PMC7187585

[B18] Sperk M. Ambikan A. T. Ray S. Singh K. Mikaeloff F. Diez R. C.. (2021). Fecal metabolome signature in the HIV-1 elite control phenotype: enrichment of dipeptides acts as an HIV-1 antagonist but a prevotella agonist. J. Virol. 95, e0047921. doi: 10.1128/JVI.00479-21 34232744 PMC8387056

[B19] Yoshinaga T. Kobayashi M. Seki T. Miki S. Wakasa-Morimoto C. Suyama-Kagitani A.. (2015). Antiviral characteristics of GSK1265744, an HIV integrase inhibitor dosed orally or by long-acting injection. Antimicrob. Agents Chemother. 59, 397–406. doi: 10.1128/AAC.03909-14 25367908 PMC4291378

[B20] Zhang W. W. Cheung P. K. Oliveira N. Robbins M. A. Harrigan P. R. Shahid A. (2018). Accumulation of multiple mutations *in vivo* confers cross-resistance to new and existing integrase inhibitors. J. Infect. Dis. 218, 1773–1776. doi: 10.1093/infdis/jiy428 30010985

