# Supplementary material for: Broad synergistic antiviral efficacy between a novel elite controller-derived dipeptide and antiretrovirals against drug-resistant HIV-1
Source: Front Cell Infect Microbiol. 2024 Jun 10;14:1334126. doi: 10.3389/fcimb.2024.1334126 (PMC11194349; doi:10.3389/fcimb.2024.1334126)
Supplement: Supplementary file 1 [file DataSheet_1.docx]

**Supplementary Figure 1. Membrane integrity assay of WG-am or VQ-am and ssON.** LDH assays were performed on TZM.bl cells after 48h. Cells were treated with WG-am (1mM) in combination with RAL (1µM), TDF (10µM), DRV (100nM), and EFV (1µM). Non-treated cells were used as cell viability control and were set as 100% viability and the values below 80% (red dotted line) were regarded as reduced viability based on SD of non-treated cells. Data shown represent the mean of two independent experiments. DMSO 10% was used as toxicity control.

**Supplementary Table 1.** **Drug resistant isolates**. The isolates were provided by NIH with information about the respective resistance mutations and IC50 fold increase to the selected antivirals. PI: protease inhibitors, INSTI: integrase strand transfer inhibitors; NRTI: nucleotide analog reverse transcriptase inhibitors; NNRTI: non-nucleoside analog RTI. Tenofovir disoproxil fumarate (TDF), raltegravir (RAL), efavirenz (EFV) and darunavir (DRV).

**Supplementary Table 2. Combination index (CI), EC50, Loewe synergy score calculations of WG-am combined with RAL, TDF, DRV, and EFV against isolates with resistance to integrase strand transfer inhibitors (INSTI).** The CI at different effective doses (ED) was determined using Calcusyn software. The ZIP synergy score was calculated using SynergyFinder 3.0 software.

**Supplementary Table 3A. Combination index (CI), EC50, Loewe synergy score calculations of WG-am combined with RAL, and EFV against isolates with resistance to protease inhibitors (PI).** The CI at different effective doses (ED) was determined using Calcusyn software. The ZIP synergy score was calculated using SynergyFinder 3.0 software.

**Supplementary Table 3B. Combination index (CI), EC50, Loewe synergy score calculations of WG-am combination with TDF, and DRV against isolates with resistance to protease inhibitors (PI).** The CI of the compounds at different effective doses (ED) was determined using Calcusyn software. The ZIP synergy score was calculated using SynergyFinder 3.0 software.

**Supplementary Table 4. Combination index (CI), EC50, Loewe synergy score calculations of WG-am combined with RAL, TDF, DRV, and EFV against isolates with resistance nucleoside reverse transcriptase inhibitor (NRTI).** The CI at different effective doses (ED) was determined using Calcusyn software. The ZIP synergy score was calculated using SynergyFinder 3.0 software.

**Supplementary Table 5A. Combination index (CI), EC50, Loewe synergy score calculations of WG-am combinated with RAL, and EFV against isolates with resistance to non-nucleoside reverse transcriptase Inhibitor (NNRTI).** The CI at different effective doses (ED) was determined using Calcusyn software. The ZIP synergy score was calculated using SynergyFinder 3.0 software.

**Supplementary Table 5B. Combination index (CI), EC50, Loewe synergy score calculations of WG-am combined with TDF, and DRV against isolates with resistance to non-nucleoside reverse transcriptase inhibitor (NNRTI).** The CI at different effective doses (ED) was determined using Calcusyn software. The ZIP synergy score was calculated using SynergyFinder 3.0 software.
